# Modulation of the balance of fatty acid production and secretion is crucial for enhancement of growth and productivity of the engineered mutant of the cyanobacterium *Synechococcus elongatus*

**DOI:** 10.1186/s13068-016-0506-1

**Published:** 2016-04-23

**Authors:** Akihiro Kato, Kazuhide Use, Nobuyuki Takatani, Kazutaka Ikeda, Miyuki Matsuura, Kouji Kojima, Makiko Aichi, Shin-ichi Maeda, Tatsuo Omata

**Affiliations:** Graduate School of Bioagricultural Sciences, Nagoya University, Nagoya, 464-8601 Japan; Institute for Advanced Biosciences, Keio University, Tsuruoka, Yamagata 997-0052 Japan; Department of Biological Chemistry, Chubu University, Kasugai, 487-8501 Japan; Japan Science and Technology Agency, CREST, Tokyo, Japan; Laboratory for Metabolomics, RIKEN Center for Integrative Medical Sciences, Yokohama, 230-0045 Japan; Laboratory of Molecular Plant Physiology, Graduate School of Bioagricultural Sciences, Nagoya University, Furo, Chikusa, Nagoya, 464-8601 Japan

**Keywords:** Cyanobacteria, Biofuel production, Free fatty acids, O-antigen

## Abstract

**Background:**

Among the three model cyanobacterial species that have been used for engineering a system for photosynthetic production of free fatty acids (FFAs), *Synechococcus elongatus* PCC7942 has been the least successful; the FFA-excreting mutants constructed from this strain could attain lower rates of FFA excretion and lower final FFA concentrations than the mutants constructed from *Synechocystis* sp. PCC6803 and *Synechococcus* sp. PCC7002. It has been suggested that *S. elongatus* PCC7942 cells suffer from toxicity of FFA, but the cause of the low productivity has remained to be determined.

**Results:**

By modulating the expression level of the acyl–acyl carrier protein thioesterase and raising the light intensity during cultivation, FFA secretion rates comparable to those obtained with the other cyanobacterial species were attained with an engineered *Synechococcus elongatus* mutant (dAS1T). The final FFA concentration in the external medium was also higher than previously reported for other *S. elongatus* mutants. However, about 85 % of the total FFA in the culture was found to remain in the cells, causing severe photoinhibition. Targeted inactivation of the *wzt* gene in dAS1T, which gene manipulation was previously shown to result in loss of the hydrophilic O-antigen layer on the cell surface, increased FFA secretion, alleviated photoinhibition, and lead to 50 and 45 % increase in the final cell density and the total amount of FFA in the culture (i.e., the sum of the cellular and extracellular FFA), respectively. The average rate of production of total FFA by the culture of the ∆*wzt* strain was 2.7 mg L^−1^ h^−1^, being five times higher than those reported for *Synechocystis* sp. PCC 6803 and comparable to the rates of triacylglycerol production in green algae.

**Conclusion:**

*Synechococcus elongatus* PCC7942 has larger capacity of FFA production than *Synechocystis* sp. PCC6803 but accumulates most of the product in the cell because of the imbalance of the rates of FFA production and secretion. This causes severe photoinhibition and exerts adverse effects on cell growth and FFA productivity. Enhancement of FFA secretion would be required to fully exploiting the capacity of FFA production for the purpose of biofuel production.

**Electronic supplementary material:**

The online version of this article (doi:10.1186/s13068-016-0506-1) contains supplementary material, which is available to authorized users.

## Background

Production of free fatty acids (FFAs) using genetically engineered cyanobacteria is thought to be a promising method of production of renewable biofuels, because FFAs are excreted into the medium and as a result, the two energy-intensive steps in microbial biofuel production, i.e., recovery of the cells from the growth medium and extraction from the cells of the photosynthetic product, can be omitted [1–3]. The highest reported yield of FFA production to date is a final FFA concentration of 197 mg L^−1^ achieved with an average production rate of 0.438 mg L^−1^ h^−1^ obtained with an engineered strain of *Synechocystis* sp. PCC 6803 [1]. However, the yield of cyanobacteria-based FFA production is much lower than that reported for algae-based triacylglycerol (TAG) production [4].

FFA production can be achieved in cyanobacteria by two means: (1) inactivation of the endogenous gene encoding acyl-ACP synthetase (Aas) [5], which recycles FFA via esterification to ACP, and (2) introduction of a foreign thioesterase(s) having the capacity to hydrolyze acyl–acyl carrier protein (ACP) [1]. Aas-deficient mutants expressing truncated thioesterase from *E. coli* (‘TesA) have been constructed from three strains of model cyanobacteria, *Synechocystis* sp. PCC6803, *Synechococcus* sp. PCC7002, and *Synechococcus elongatus* PCC7942. In *Synechocystis* sp. PCC6803, an extracellular FFA concentration of 83.6 mg L^−1^ with an average FFA excretion rate of 0.17 mg^−1^ L^−1^ h^−1^ was attained by simply introducing *‘tesA* and inactivating *aas*, and the FFA productivity was further improved to attain the extracellular FFA level of 197 mg L^−1^ (see above) by additional gene manipulation(s) aimed at inactivation of the PHB biosynthesis pathway, weakening of the peptidoglycan layer, overexpression of acetyl-CoA carboxylase, and expression of thioesterases from various sources other than *E. coli* [1]. In *Synechococcus* sp. PCC 7002 and *Synechococcus elongatus* PCC 7942, the *‘tesA*-expressing *aas*-deficient mutants showed relatively low FFA productivity, attaining extracellular FFA concentrations of 40 and 49.3 mg L^−1^ with average excretion rates of 0.083 and 0.103 mg L^−1^ h^−1^, respectively [2, 3]. While FFA production by the *Synechococcus* sp. PCC7002 mutant was increased to 131 mg L^−1^ with an average excretion rate of 0.27 mg L^−1^ h^−1^ by overexpression of Rubisco [2], similar gene manipulation failed to increase FFA production by *S. elongatus* PCC7942 mutants, leading to a hypothesis that adverse effects of FFA on cellular activities are limiting FFA production in the latter strain [6]. Although the molecular basis of FFA toxicity remains to be determined, these results indicated that different factors limit FFA production in different cyanobacterial species [2].

In an attempt to increase FFA production *per*-*cell* of *S. elongatus* PCC7942 mutants, Kato et al. [7] overexpressed *‘tesA* while minimizing cell growth by nitrogen limitation. This, however, killed the cells, suggesting that overproduction of FFAs during growth limitation is lethal. A fortuitously obtained pseudorevertant capable of growth under these conditions was found to overexpress an RND-type efflux system having the capacity of FFA export and shown to excrete FFAs at an average rate of 0.23 mg L^−1^ h^−1^, which was twice as high as the rate that had been attained in *S. elongatus* PCC7942 [7]. The pseudorevertant nevertheless could not sustain growth for >200 h and the final FFA concentration was only 45 mg L^−1^, being similar to the previously reported values. In this study, we show that modulation of the balance of FFA production in the cell and FFA export out of the cell is essential to further increase the FFA productivity. In spite of a high FFA excretion rate thus attained, which is similar to the highest rate ever reported in cyanobacterial mutants, the mutant cells are shown to retain most of the FFAs intracellularly and suffer from photoinhibition. Possible strategies to exploit the unexpectedly large capacity of FFA synthesis found in *S. elongatus* PCC7942 for biofuel production are discussed.

## Results

### Improvement of FFA productivity by attenuation of ‘tesA expression

**Fig. 1:**
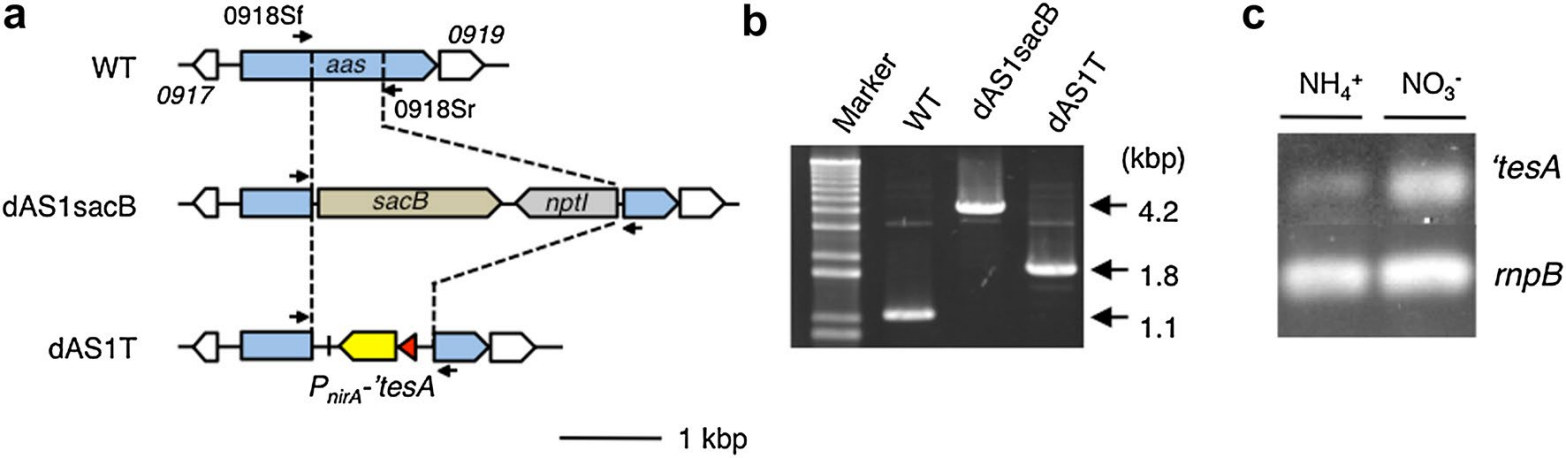
Construction of the FFA-producing strains from *S. elongatus*. **a** Map of the *aas* region of the genomes of WT and the mutants. A transcriptional fusion of the *nirA* promoter from *S. elongatus* (shown in *red*) and the *‘tesA* coding sequence from *E. coli* (shown in *yellow*) was used to replace an intragenic 662 bp fragment of the *aas* gene via the marker-exchange eviction method to construct dAS1T. *Arrows* above the map indicate the primers used for PCR analysis in **b**. **b** PCR analysis of the *aas* region of WT and the mutants. **c** Semi-quantitative RT-PCR analysis of the *‘tesA* transcript in dAS1T, showing the effect of nitrogen source. Total RNA was extracted from dAS1T cells at *t* = 48 h and subjected to the analysis, using the *rnpB* transcript as a control. Cycle numbers for PCR were 24 and 26 for ‘*tesA* and *rnpB*, respectively

**Fig. 2:**
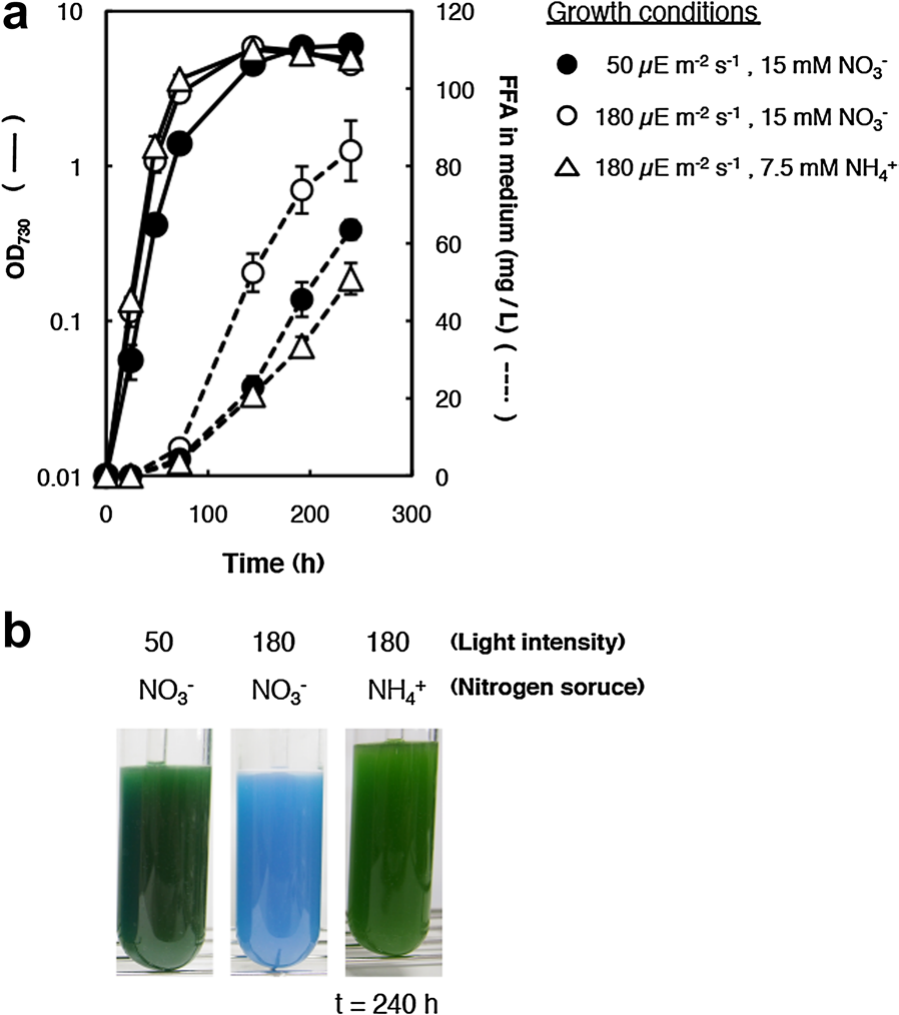
Effects of light intensity and nitrogen source on growth and FFA production. **a** Growth and FFA production of dAS1T. Cells grown in nitrate-containing medium under low light (50 μE m^−2^ s^−1^) were harvested and transferred to nitrate or ammonium-containing medium at *t* = 0 and incubated under high light (180 μE m^−2^ s^−1^) or low light (50 μE m^−2^ s^−1^). *Solid lines* optical density at 730 nm (OD_730_); *dotted lines* FFA concentrations in medium. *Closed circles* cells grown in nitrate-containing medium under low light (*n* = 3); *Open circles* cells grown in nitrate-containing medium under high light (*n* = 6); *Open triangles* cells grown in ammonium-containing medium under high light (n = 3). Data shown are the mean ± SE from the biological replicates. **b** Appearance of the cultures at *t* = 240 h, showing the growth defect of dAS1T in nitrate-containing medium under high light after prolonged cultivation

In the FFA-producing strain dAS1T, the *‘tesA* coding sequence was transcriptionally fused to the nitrogen-regulated promoter (*P*_*nirA*_) of the nitrate assimilation operon (*nirA*-*nrtABCD*-*narB*) and introduced into the *aas* locus (Fig. 1a, b). While *P*_*nirA*_ is inactive in the presence of ammonium in growth media, it sustains low but robust expression of the downstream genes when the cells are grown in media containing sufficient amounts of nitrate as the nitrogen source [8, 9]. Semi-quantitative RT-PCR analysis of the *‘tesA* transcript confirmed the nitrogen-responsive regulation of the *P*_*nirA*_ promoter in dAS1T (Fig. 1c). Since *P*_*nirA*_ is most active under constantly nitrate-limited growth conditions, which can be achieved in a chemostat or by forcing nitrate transporter-less mutants to grow slowly in media containing high concentrations of nitrate that allow for slow, passive diffusion of nitrate into the cell [8, 9], Kato et al. [7] once introduced the same *P*_*nirA*_-*‘tesA* transcriptional fusion to the *aas* locus in a nitrate transporter-less *S. elongatus* mutant to maximize FFA production while minimizing cell growth [7]. However, the resultant mutant (dAS2T) died under the ‘*tesA*-inducing conditions in nitrate-containing media, showing that overexpression of ‘*tesA* is lethal. Unlike dAS2T, dAS1T could grow in the nitrate-containing medium, presumably due to attenuation of *‘tesA* expression (Fig. 2a). Under low-light conditions (50 μE m^−2^ s^−1^) in the nitrate-containing medium, dAS1T secreted FFA at an average rate of 0.27 mg L^−1^ h^−1^ (Fig. 2a, filled circles), attaining a FFA concentration in the medium of 64 mg L^−1^ in 240 h. These figures were higher than those obtained previously under similar growth conditions in a *S. elongatus* PCC 7942 *aas* mutant carrying a *P*_*trc*_-driven *‘tesA*: an average excretion rate of 0.1 mg L^−1^ h^−1^ and a final FFA concentration of 49 mg L^−1^ obtained in 480 h [3]. These results seemed to suggest that attenuation of *‘tesA* expression contributed to the improved FFA productivity of dAS1T.

### Enhanced FFA production under high-light conditions and its toxic effects

The FFA secretion rate of dAS1T in the nitrate-containing medium was increased by raising the light intensity to 180 μE m^−2^ s^−1^ (Fig. 2a, open circles); the average FFA excretion rate of 0.35 mg L^−1^ h^−1^ was thus attained, but the cells died in 240 h of cultivation with complete loss of chlorophyll, showing blue coloration due to the remaining phycobiliproteins (Fig. 2b). When grown on ammonium under the high-light conditions, dAS1T showed slower excretion of FFA and remained green (Fig. 2a triangles and b). These results showed that the high-light condition *per se* was not toxic to the cells. Using an *aas* single mutant of *S. elongatus* (dAS1), Takatani et al. [10] showed that production of FFAs renders the cyanobacterial cells sensitive to photodamage [10]. Since dAS1 did not show such a severe growth phenotype as was observed with dAS1T [10], it was deduced that the increased FFA production due to expression of ‘TesA lead to the growth inhibition in dAS1T under the high-light conditions.

### Toxicity of FFA overproduction is alleviated by impairment of O-antigen production

**Fig. 3:**
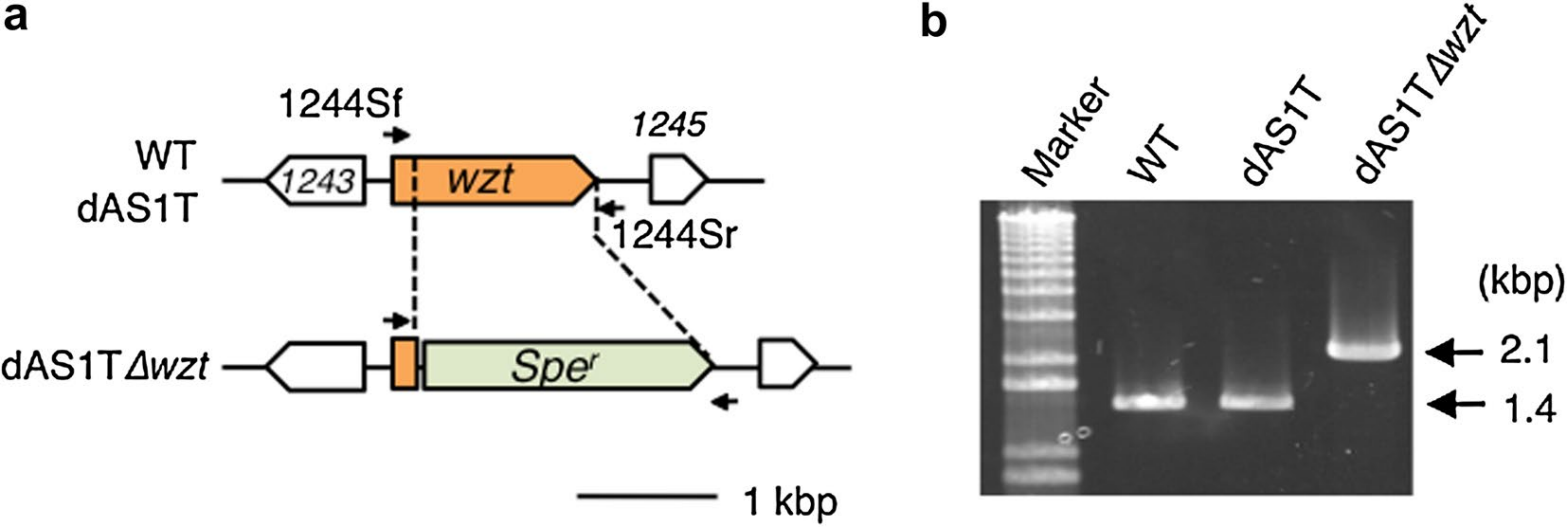
Construction of the *wzt*-deficient mutant from dAS1T. **a** Map of the *wzt* region of the genomes of WT, dAS1T, and dAS1T∆*wzt*. *Arrows* above the map indicate the primers used for PCR analysis in **b**. **b** PCR analysis of the *wzt* region of the genomes of WT, dAS1T, and dAS1T∆*wzt*

The adverse effects of enhanced FFA production on growth of the dAS1T cells suggested that intracellular accumulation of FFA was toxic. To further improve the FFA productivity of dAS1T, we attempted to facilitate passive diffusion of FFAs out of the cells by modification of the cell surface structure. Cyanobacteria as well as other gram-negative bacteria have inner (cytoplasmic) and outer membranes, with lipopolysaccharide (LPS) being a major component of the latter. The polysaccharide portion of LPS, consisting of the O-antigen chain and the core oligosaccharide complex, comprises the hydrophilic layer on the cell surface, which has been shown to act as a diffusion barrier to protect bacterial cells against extracellularly added FFAs [11, 12]. This suggested that elimination of O-antigen, which comprises the outermost and the largest portion of the polysaccharide chain of LPS, might help release of FFAs out of the FFA-producing cyanobacterial cells. We hence constructed in this study an O-antigen deficient mutant from dAS1T by inactivating the *wzt* gene involved in the export of the O-antigen unit across the cytoplasmic membrane [13]. Impairment of O-antigen production was reported to render the cell surface of *S. elongatus* hydrophobic and to confer the cells the resistance against grazing by amoebae, without exerting any negative effects on growth [13]. The gene was successfully inactivated (Fig. 3) and the resulting mutant showed the autoflocculation phenotype as previously reported [13].

**Fig. 4:**
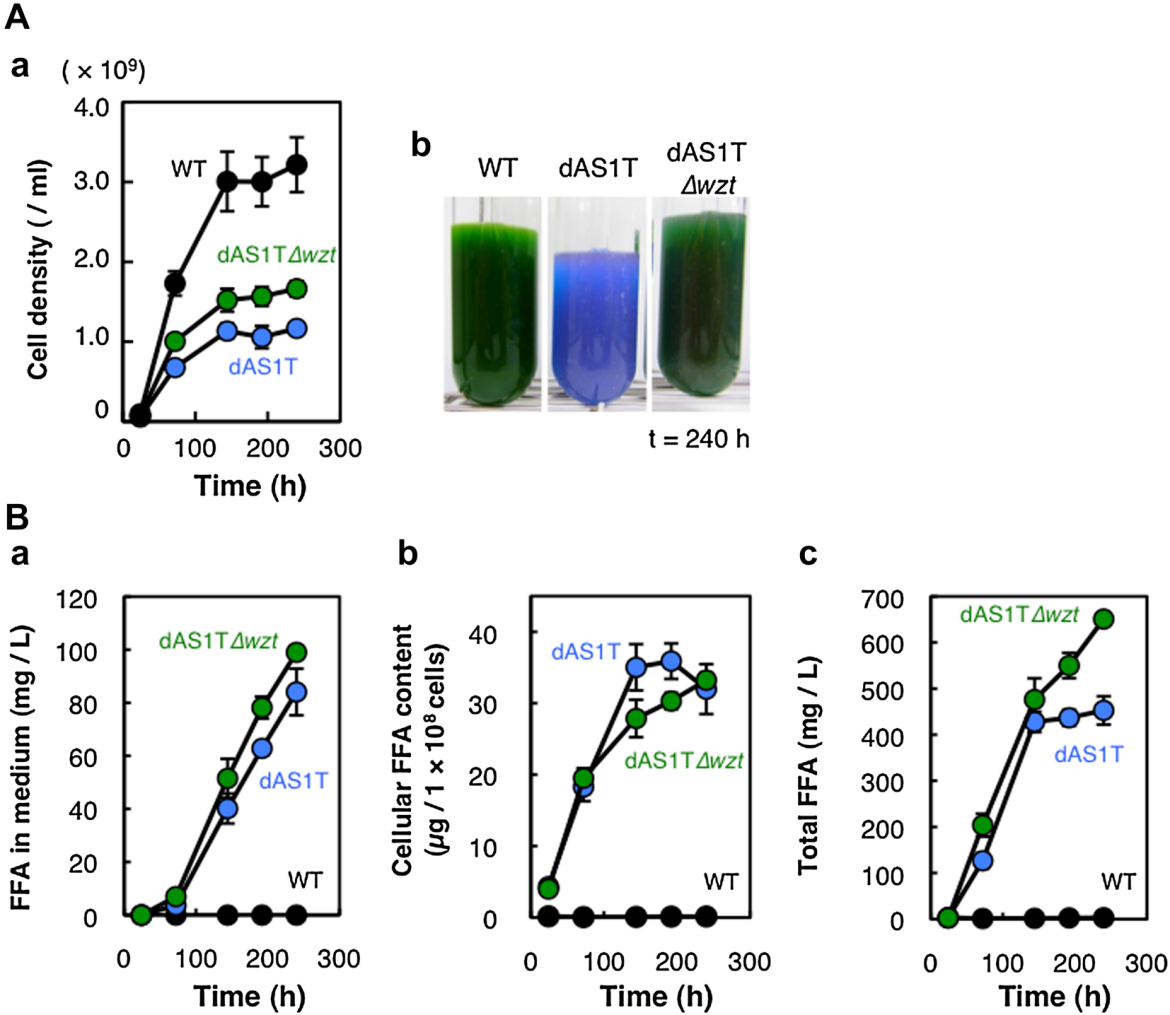
Effects of *wzt* deficiency on growth and FFA production. WT, dAS1T, and dAS1T∆*wzt* cells grown under low light (50 μE m^−2^ s^−1^) in nitrate-containing medium were inoculated at time zero into new nitrate-containing medium and incubated under the high-light conditions (180 μE m^−2^ s^−1^). **A** Cell density measured at designated times (*a*) and appearance of the cultures at *t* = 240 h (*b*). **B** Changes in extracellular FFA concentration (*a*), cellular FFA content (*b*), and the sum of cellular and extracellular FFA in the cultures (*c*). *Black circles* WT; *blue circles* dAS1T; *green circles* dAS1T∆*wzt*. Data shown are the mean ± SE from biological triplicates

Figure 4Aa compares growth of dAS1T and dAS1T∆*wzt* strains with that of WT under the high-light conditions with nitrate as the nitrogen source. Growth of dAS1T was much slower than that of WT, with the final cell density being about 35 % of the WT level. The dAS1T∆*wzt* strain grew faster than the parental dAS1T strain and attained a final cell density corresponding to about 50 % of WT. Unlike the dAS1T strain, which completely lost chlorophyll in 240 h, the dAS1T∆*wzt* cells retained chlorophyll after 240 h of cultivation (Fig. 4Ab). Thus, the loss of O-antigen alleviated the toxic effects exerted by FFA production under the high-light conditions. It should be noted, however, that the dAS1T∆*wzt* cultures turned into blue in coloration in 264 h (not shown), indicating that the “early-death” phenotype of the dAS1T strain was partially rescued by the loss of O-antigen.

Measurements of FFA concentration in the medium during cultivation of the FFA-producing strains revealed a small increase in the rate of FFA excretion in the dAS1T∆*wzt* strain compared with the parental dAS1T strain (Fig. 4Ba). The average rate of FFA excretion during the 240 h of cultivation was 0.41 mg L^−1^ h^−1^ in dAS1T∆*wzt*, which was about 15 % higher than that in dAS1T and close to the highest rate achieved in cyanobacteria: 0.438 mg L^−1^ h^−1^ obtained with an engineered strain of *Synechocystis* sp. PCC 6803 [1]. The FFA concentration in the external medium of dAS1T∆*wzt* reached 100 mg L^−1^, which was 12 % higher than that attained by dAS1T.

### O-antigen deficiency results in improvement of FFA productivity

In both dAS1T and dAS1T∆*wzt*, the cellular FFA content was initially low and was increased during growth (Fig. 4Bb). In the initial phase of growth (up to *t* = 72 h), cellular FFA increased in a similar manner in dAS1T and dAS1T∆*wzt*, but the increase was slower thereafter in dAS1T∆*wzt* than in dAS1T. At *t* = 144 h, cellular contents of FFAs were calculated to be 175 and 155 mg per mL of cell volume for dAS1T and dAS1T∆*wzt*, respectively. The total amount of FFAs in the culture, as calculated from the data shown in Fig. 4Aa, Ba, b, increased in a similar manner in the two strains until they reached the stationary phase of growth at *t* = 144 h (Fig. 4Bc). During the stationary phase, there was essentially no increase in total FFAs in the dAS1T culture. The continued increase of extracellular FFAs (Fig. 4Ba) and a decline in cellular FFA content (Fig. 4Bb) in the dAS1T cultures during the stationary phase suggested that FFAs are released from dead cells in the last stage of cultivation. Unlike dAS1T cells, the cells of dAS1T∆*wzt* continued FFA production during the stationary phase to attain the total FFA level of 650 mg L^−1^, which was about 45 % higher than that in the dAS1T cultures. The average rate of production of total FFA by the dAS1T∆*wzt* culture was 2.7 mg L^−1^ h^−1^, being comparable to some of the examples of the rate of TAG production by green algae [14]. Although dAS1T∆*wzt* attained the highest level of extracellular FFA ever reported for the FFA-producing strains obtained from *S. elongatus* [3], it should be noted that most of FFA in the culture was retained in the cell.

**Fig. 5:**
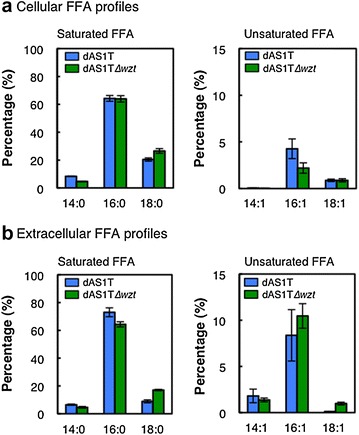
Cellular and extracellular FFA profiles of the FFA-producing strains. dAS1T and dAS1T∆*wzt* cells were grown under the high-light conditions (180 μE m^−2^ s^−1^) in nitrate-containing medium for 192 h. **a** Cellular and **b** extracellular FFA compositions were analyzed by LC–MS. *Left panel* saturated FFA (14:0, 16:0 and 18:0); *right panel* unsaturated FFA (14:1, 16:1, 18:1). *Blue bars* dAS1T; *green bars* dAS1T∆*wzt*. Data shown are the mean ± SE from biological triplicates

In dAS1T∆*wzt* as well as in dAS1T, palmitic acid (16:0) was the major constituent of both the cellular and extracellular pools of FFAs, comprising >60 % of the total FFAs (Fig. 5). Together with stearic acid (18:0) and myristic acid (14:0), the saturated fatty acids comprised about 90 % and 85 % of the cellular and extracellular FFA pools, respectively. As previously observed [5, 7], the relative contents of palmitoleic acid (16:1) and myristoleic acid (14:1) were significantly higher in the extracellular FFA pool than in the cellular pool in both of the strains (Fig. 5). There was, however, no significant difference between the dAS1T and dAS1T∆*wzt* strains in their cellular and extracellular FFA profiles.

### O-antigen deficiency results in protection of photosystems

**Fig. 6:**
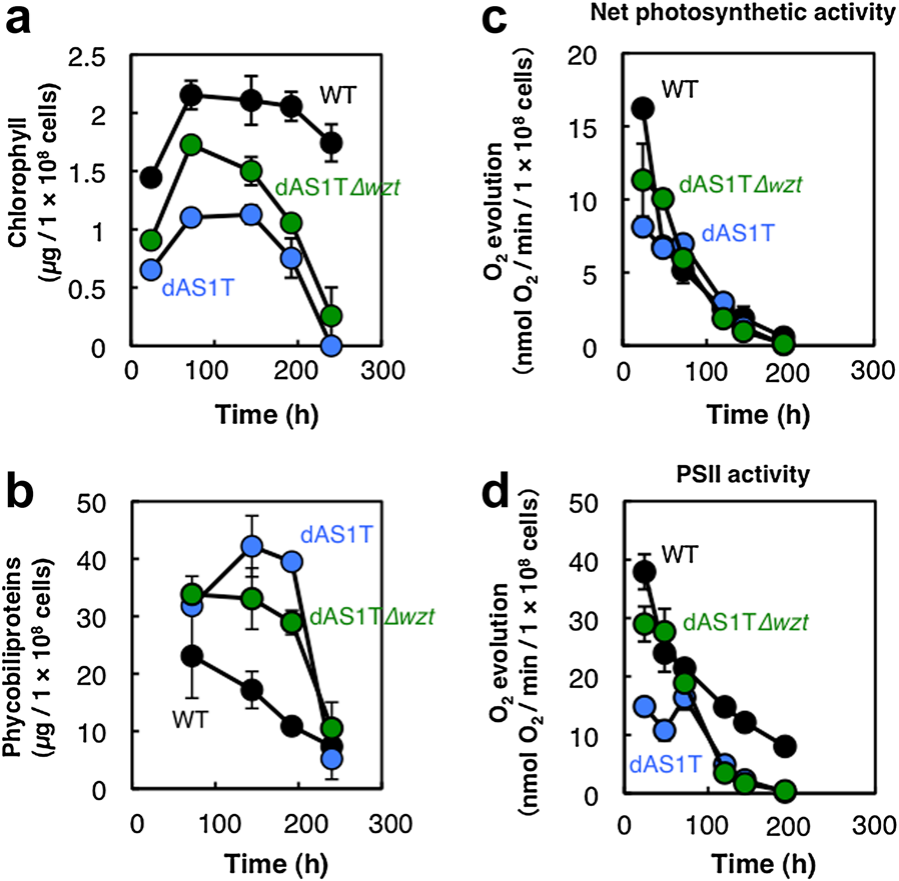
Changes in the cellular pigment contents, and photosynthetic activities during cultivation. WT, dAS1T, and dAS1T∆*wzt* cells were grown under high light (180 μE m^−2^ s^−1^) in nitrate-containing medium. **a**, **b** Chlorophyll and phycobiliprotein contents, respectively. Data are the mean ± SE from the same biological triplicates shown in Fig. 4. **c**, **d** Activities of photosynthetic O_2_ evolution measured with CO_2_ and 1 mM 1,4-benzoquinone as the electron acceptor, respectively, under illumination at 1000 μE m^−2^ s^−1^. Data shown are the mean ± SE from biological triplicates. *Black circles* WT; *blue circles* dAS1T; *green circles* dAS1T∆*wzt*

Time courses of the changes of pigment contents and photosynthetic activities of the cells of WT, dAS1T, and dAS1T∆*wzt* are shown in Fig. 6. The chlorophyll content of WT cells was increased by 50 % during the logarithmic phase of growth and then remained at a high level. The chlorophyll content of dAS1T cells corresponded to 35–55 % of the WT level during cell growth, and was rapidly decreased during the stationary phase, resulting in complete loss of the pigment in 240 h of cultivation. The chlorophyll content of the dAS1T∆*wzt* cells was higher than that of the dAS1T cells during the logarithmic phase of growth, suggesting that inactivation of *wzt* alleviated the adverse effects of FFA production on the photosystems. Unlike the content of chlorophyll, the content of phycobiliproteins in dAS1T and dAS1T∆*wzt* cells was much higher than the wild-type level.

Since the cellular chlorophyll content of the FFA-producing strains greatly changed during the cultivation, the photosynthetic activity was expressed not on a per-chlorophyll basis, but a per-cell basis. (Fig. 6c, d). In each of the strains, the rate of CO_2_-dependent O_2_ evolution, which represents the overall activity of photosynthetic CO_2_ fixation, was highest in the beginning of cultivation and declined to an insignificant level in 192 h (Fig. 6c). The activities in the WT and the FFA-producing strains were similar in the late-logarithmic to stationary phase of growth, but in the beginning of cultivation WT showed the highest activity and the dAS1T showed the lowest activity. The PSII electron transport activity of WT, which was measured as the rate of O_2_ evolution using 1,4-benzoquinone as the electron acceptor, also declined from a high level in the beginning to a low level during the course of cultivation, but unlike the CO_2_ fixation activity about 20 % of the initial activity remained in the cell even after 192 h of cultivation (Fig. 6d). By contrast, the PSII activity of dAS1T was low from the beginning and sharply declined to a negligible level in 192 h. The PSII activity of dAS1T∆*wzt* cells was twice as high as that of the dAS1T cells in the earlier stage of growth but decreased sharply as in dAS1T to a negligible level in 192 h. These results showed that inactivation of the *wzt* gene mitigated the adverse effects of FFA production in the dAS1T cells.

**Fig. 7:**
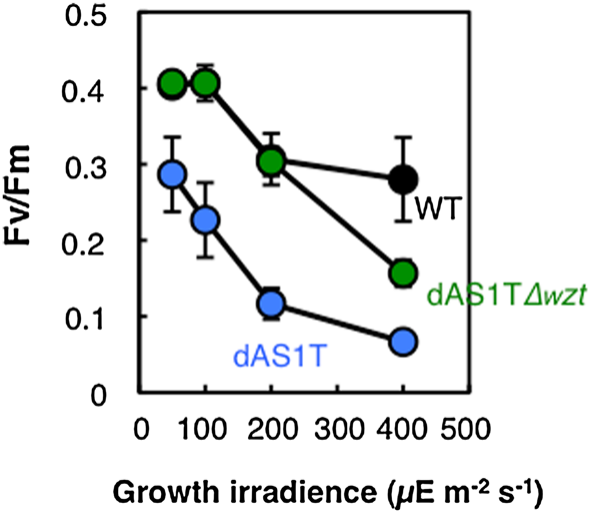
Effects of growth irradiance on the photosynthetic yield of WT and the FFA-producing strains. WT, dAS1T, and dAS1T∆*wzt* cells grown under low light (50 μE m^−2^ s^−1^) in nitrate-containing medium were transferred to new nitrate-containing medium and cultivated for 36 h under continuous illumination at the designated light intensities, provided by white-light emitting diode lamps (VBL-SL150-LL, Valore). *Black circles* WT; *blue circles* dAS1T; *green circles* dAS1T∆*wzt*. Data shown are the mean ± SE from biological triplicates

Figure 7 shows the effects of growth irradiance on the photosynthetic yield of PSII as determined by measuring the Fv/Fm ratio. The Fv/Fm ratio of dAS1T was lower than that of WT even when grown under low-light conditions (50 μE m^−2^ s^−1^) and decreased more sharply than that of WT as the light intensity during growth was increased (Fig. 7). The Fv/Fm ratio of dAS1T∆*wzt* was essentially the same as that of WT up to a growth irradiance of 200 μE m^−2^ s^−1^. At 400 μE m^−2^ s^−1^, Fv/Fm declined to a level lower than that of WT, but was much higher than that of dAS1T. Thus, the inactivation of the *wzt* gene was effective in protecting PSII from photoinhibition.

## Discussion

Among the FFA-producing cyanobacterial mutants constructed to date, the SD277 strain of *Synechocystis* sp. PCC 6803 is the most productive. Final FFA concentration of about 200 mg L^−1^ in the external medium was attained with an average secretion rate of 0.44 mg L^−1^ h^−1^ [1]. *Synechococcus elongatus* PCC 7942 mutants engineered for FFA production have been much less productive (summarized in Ruffing [2]), but the dAS1T mutant constructed in this study shows a relatively high rate of FFA excretion (0.35 mg L^−1^ h^−1^) when cultivated under high-intensity light (Figs. 2, 4). The final FFA concentration in the medium, however, was less than half of that attained by SD277, because of the early death of the dAS1T cells (Figs. 2, 4). Since elimination of the O-antigen layer from the cell surface resulted in a small decrease and increase in cellular and extracellular FFA, respectively, and significantly enhanced cell growth (Fig. 4), it is deduced that accumulation of excessive FFA in the cell was the cause of the early death of dAS1T. Unlike the SD277 mutant of *Synechocystis* sp. PCC 6803, which excretes about 95 % of the total FFA into the medium [1], the dAS1T cells carry about 85 % of the total FFA in the culture (Fig. 4B). These results indicate that the FFA productivity of the *S. elongatus* mutant is limited by the slow permeation of FFA across the cell envelope.

Taking into account the FFAs associated with in the cells, the total FFA contents in the cultures of the SD277 mutant of *Synechocystis* sp. PCC 6803 and the ∆*wzt* derivative of dAS1T are calculated to be 210 and 650 mg L^−1^, respectively, at the end of cultivation. Thus, *S. elongatus* PCC 7942 has a much greater capacity for fatty acid biosynthesis than *Synechocystis* sp. PCC 6803. The average rate of total FFA production by the *S. elongatus* PCC 7942 mutant is calculated to be 2.7 mg L^−1^ h^−1^, which is comparable to the rates of TAG production reported for the green algae, *Chlorella vulgaris,Chlorella emersonii*, and *Nannochloropsis gaditana* [15, 16]. Since most cyanobacteria including *S. elongatus* PCC 7942 accumulate glycogen as the storage material under the conditions of N limitation [17–19], it has been supposed that cyanobacteria are not well suited for lipid production. The present results, however, demonstrate that certain species of cyanobacteria have a large potential as a source of lipids to be used as biodiesel.

Although inactivation of the *wzt* gene in dAS1T increased the FFA secretion rate to 0.41 mg^−1^ L^−1^ and delayed accumulation of FFAs in the cell (Fig. 4), the cellular FFA content of the ∆*wzt* strain was increased to a level as high as that in the parental strain after 240 h of cultivation. The final concentration of FFA in the external medium was about 100 mg L^−1^, being about half of that attained by the *Synechocystis* SD277 mutant in 450 h. The cultures of the ∆*wzt* strain were green at *t* = 240 h (Fig. 4A), but assumed a blue coloration after 264 h of cultivation (not shown), indicating that the early-death phenotype of the dAS1T strain was only partially rescued by the inactivation of the *wzt* gene. To further increase the productivity of the extracellular FFA, the rate of FFA excretion needs to be increased. To this end, the RND-type export system having the capacity of FFA excretion, which was recently identified in *S. elongatus* PCC 7942 [7], would be useful. Given the large capacity for FFA production found in the *S. elongatus* 7942 mutants, it would also be necessary to introduce multiple export systems for FFAs, including those identified in other bacterial species [20–22]. Given the presence of the intracellular membrane system (the thylakoids) in the cyanobacterial cell, correct targeting of the FFA exporters to the cytoplasmic membrane would be critical particularly in the attempts to overexpress the transporters. The other possible strategy to increase the FFA in the external medium is to regulate FFA production in the cell during the stationary phase of growth. Without cell division, accumulation of FFA to a lethal level for the cell is inevitable if the rate of FFA production exceeds the rate of its excretion. This approach will sacrifice the large capacity of FFA production in the *S. elongatus* cell, but by balancing the rates of FFA production and excretion, sustained excretion of FFA for longer periods of time will be possible. Upon successful functional expression of FFA exporter(s), FFA production can be deregulated to meet the increased rate of FFA excretion.

Using an *aas* single mutant of *S. elongatus* PCC 7942 (dAS1), Takatani et al. [10] showed that deacylation of membrane lipids is activated under high-light conditions [10]. PSII is unstable in dAS1 and is much more sensitive to high light than in WT, presumably due to accumulation of FFAs and/or lysolipids [10]. As in dAS1, the PSII activity of dAS1T is hypersensitive to high light, but elimination of the O-antigen layer effectively protects PSII from photoinhibition (Fig. 7), suggesting that intracellular accumulation of FFA is the major cause of destabilization of PSII. FFAs act as surfactants and are known to have various adverse effects on cellular activities [23]. dAS1T produces FFAs not only from membrane lipids but from acyl-ACP via the action of the ‘TesA thioesterase. Since the high-light conditions stimulate photosynthesis and the synthesis of acyl-ACP, production of FFAs from acyl-ACP would also be stimulated under high-light conditions. dAS1T is therefore supposed to be suffering more seriously than dAS1 from various biochemical and biophysical defects including photoinhibition of PSII under the high-light conditions. It may be hence not surprising that dAS1T cells die under the high-light conditions in an unusual manner, i.e., with complete degradation of chlorophyll but not of phycobiliproteins, giving rise to the blue coloration of the culture (Figs. 2b, 4Ab). Although the inactivation of the *wzt* gene did not fully rescue the early-death phenotype of dAS1T, enhancement of cell growth, and FFA production, and protection of the activity of PSII in dAS1T∆*wzt* suggest that the O-antigen deficiency effectively decreased intracellular accumulation of FFAs. Simkovsky et al. [13] suggested that *S. elongatus* mutants defective in O-antigen production are suited for biofuel production, as they are resistant to grazing by amoebae and capable of autoflocculation without showing apparent growth defects [13]. The present results show that the loss of O-antigen brings about additional useful traits in the FFA-producing *S. elongatus* strains: facilitation of FFA release from the cells, alleviation of photoinhibition, and enhancement of growth and FFA production. Thus, all the known phenotypes resulting from O-antigen deficiency would directly contribute to the productivity, making the mutations in O-antigen production indispensable for the construction of an efficient FFA-producing system.

## Conclusions

*Synechococcus elongatus* PCC 7942 has an unusually large capacity to synthesize fatty acids, which is to be exploited for biofuel production. Currently, the high activity of FFA synthesis results in intracellular accumulation of FFA, which adversely affects cellular metabolism and eventually kills the cells. To achieve high rate production of FFA, excretion of FFA out of the cells via both passive and positive mechanisms would be required. Inactivation of the *wzt* gene is effective in facilitating FFA secretion and will be essential.

## Methods

### Strains and growth conditions

Strains and plasmids used in this study are shown in Additional file 1: Table S1. A derivative of *Synechococcus elongatus* PCC 7942 that is cured of the resident small plasmid pUH24 (strain SPc, hereafter designated simply as wild-type strain (WT) [24]) and the FFA-producing mutants were grown photoautotrophically at 30 °C under continuous illumination, which was provided by fluorescent lamps unless otherwise stated, with aeration by air supplemented with 2 % (*v/v*) CO_2_. The basal medium used was a nitrogen-free derivative of the BG11 medium described previously [25], which was supplemented with 15 mM KNO_3_ and 3.75 mM (NH_4_)_2_SO_4_ as a nitrogen source to prepare nitrate-containing medium and ammonium-containing medium, respectively. The media were buffered with 20 mM HEPES–KOH (pH 8.2). When appropriate, spectinomycin was added to the medium at 15 μg ml^−1^. Since the PSII activity of the FFA-producing strains declines sharply during stationary phase of growth, exponentially growing cells (OD_730_ = 0.5–1.0) were used to inoculate new cultures to be used for all the experiments. Optical density of the cultures was measured at 730 nm using a spectrophotometer (UV-1700, Shimadzu). Cell volume and cell number were determined using a particle counter/analyzer (CDA-1000, Sysmex).

### Construction of the FFA-producing strains

Transformation of cyanobacteria was performed as described by Williams and Szalay [26]. The FFA-producing mutant (designated dAS1T) was obtained by introduction of a truncated *‘tesA* gene from *E. coli* transcriptionally fused to *nirA* promoter (*P*_*nirA*_) from *S. elongatus* into the *aas*-deficient mutant dAS1sacB (Fig. S1 in Takatani et al. [10]). The plasmid used to introduce the *P*_*nirA*_-*‘tesA* fusion was the p∆AAST plasmid described previously [7]. dAS1sacB was transformed with p∆AAST and the mutant dAS1T was obtained by counterselection of SacB in the presence of 10 % sucrose [27]. Segregation of the alleles was confirmed by PCR analysis of the genomic DNA from selected transformants using the primers 0918Sf and 0918Sr (Additional file 2: Table S2).

### Inactivation of the wzt gene

For construction of the *wzt*-deficient mutant derived from dAS1T (dAS1T∆*wzt*), a 945-bp DNA fragment carrying the first 45 bases of the *wzt* ORF and 900 bp of its 5′ flanking sequence was amplified by PCR using the primers 1244Uf carrying an added EcoRI site and 1244Ur carrying an added BamHI site. 1827 bp of the 3′ flanking region of *wzt* was also amplified by PCR using the primers 1244Df carrying an added XbaI site and 1244Dr carrying an added HindIII site (Additional file 2: Table S2). These DNA fragments were integrated sequentially between the EcoRI and BamHI sites and the XbaI and HindIII sites of the pUC19 vector, respectively, to yield the plasmid p∆WZT. The spectinomycin resistance cassette derived from pRL463 [28] was cloned into the BamHI site of p∆WZT to yield the plasmid p∆WZTSpe^r^. dAS1T cells were transformed with p∆WZTSpe^r^ to construct the dAS1T∆*wzt* strain via replacement of most of the *wzt* ORF with the spectinomycin resistance cassette. Successful genome segregation was confirmed by PCR using the primers 1244Sf and 1244Sr (Additional file 2: Table S2).

### FFA analysis

For analysis of cellular and extracellular FFA, 5–30 ml aliquots of the cultures were centrifuged at 1700*g* for 15 min to separate the cells and the medium. The supernatant was transferred to CryoTubes (Thermo Fisher Scientific) and the cells were resuspended in 1 mL of methanol. The samples were stored at −20 °C until use. For enzymatic determination of the total concentration of FFA in the medium, the supernatant was analyzed using the Free Fatty Acid Quantification Kit (BioVision) according to the manufacture’s instruction. For analysis of the cellular and extracellular FFA profiles and the total FFA content in the cells, samples were supplemented with the internal standards (16:0[d3] and 18:0[d3]), extracted with a modified Folch method [29, 30], and analyzed by LC–MS [10, 30]. To obtain the standard curves for FFAs, methanolic solutions of FFAs were treated exactly as the samples were, including the step of extraction with the modified Folch method, and subjected to LC–MS analysis.

### Determination of Chl and phycobiliproteins

Chlorophyll (Chl) was determined as described by Mackinney [31]. For the measurement of phycobiliproteins, cells were disrupted as described by Aoki et al. [32]. The concentration of phycobiliproteins (phycocyanin and allophycocyanin) in the supernatant was determined from the absorbance at 620 and 650 nm, using the equation described by Tandeau de Marsac and Houmard [33]. Chl and PC contents were normalized to the cell number corresponding to 1.0 × 10^8^ of the cells.

### Measurement of photosynthetic activity

Photosynthetic oxygen-evolving activity of the cyanobacterial cultures was measured at 30 °C under illumination at 1000 μmol photons m^−2^ s^−1^, using a Clark-type oxygen electrode (DW1, Hansatech). The photosynthetic yield of PSII was measured using an AquaPen-C fluorometer (AP-C100, Photon Systems Instruments).

### Preparation of RNA and semi-quantitative RT-PCR

Total RNA was extracted from dAS1T cells grown with either nitrate or ammonium under high light (180 μE m^−2^ s^−1^) at *t* = 48 h, using a combination of the TRIzol^®^ Reagent (Life technologies) and the SV total RNA isolation system (Promega) as described by Kato et al. [7]. The isolated total RNA (1 μg) was used for the synthesis of cDNA, using a SuperScript™ III First-Strand Synthesis System (Life Technologies) and random primers according to the manufacture’s instruction. The obtained cDNA was used as the template for PCR analysis using the primers specific to *‘tesA* and *rnpB* (Additional file 3: Table S3).

## Additional files

10.1186/s13068-016-0506-1 Strains and plasmids used in this study. Relevant characteristics of strains and plasmids are described.
10.1186/s13068-016-0506-1 Oligonucleotides used for the construction of the mutants. Sequences are given from the 5′ end to the 3′ end.
10.1186/s13068-016-0506-1 Oligonucleotides used for semi-quantitative RT-PCR. Sequences are given from the 5′ end to the 3′ end.

## Abbreviations

FFAs: free fatty acids
TAG: triacylglycerol
Aas: acyl-ACP synthetase
ACP: acyl-acyl carrier protein
LPS: lipopolysaccharide
Spe^r^: spectinomycin resistance
WT: wild type
SPc: small-plasmid-cured
ORF: open reading frame
Chl: chlorophyll
RT-PCR: reverse transcription-PCR
